# Chylothorax in thyroid surgery: a very rare case and systematic review of the literature

**DOI:** 10.1186/s40463-016-0166-y

**Published:** 2016-10-18

**Authors:** Verena Merki, Juliane Pichler, Roland Giger, Georgios Mantokoudis

**Affiliations:** Department of Otorhinolaryngology-Head & Neck Surgery, Inselspital, Bern University Hospital, and University of Bern, 3010 Bern, Switzerland

**Keywords:** Thyroidectomy, Complication, Chylothorax, Chyle leak

## Abstract

**Background:**

Chylothorax is a very rare but major complication in thyroid surgery and should be apparent to clinicians in this field.

**Case presentation:**

We report a case with chylothrax after thyroid surgery in our department that drew our attention.

**Methods:**

Systematic review of the literature to evaluate the incidence and the contributing factors of chylothorax after thyroid surgery. Database (PubMed) and hand searches to identify patients with thyroid surgery and postoperative chylothorax. Keywords included chylothorax, thyroidectomy, thyroid surgery and complications. Two independent reviewers screened studies against inclusion and exclusion criteria. Patient characteristics, risk factors, symptoms, treatments and etiopathogenesis were investigated.

**Results:**

We identified 13 articles in the literature describing 19 patients with chylothorax after thyroidectomy and described our own case. Ninety percent of the patients underwent thyroidectomy for thyroid cancer. Sixteen patients (80 %) underwent thyroidectomy with at least a left lateral neck dissection, 2 patients (10 %) underwent thyroidectomy with sternotomy, and in the remaining 2 patients (10 %), thyroidectomy with lateral neck dissection on both sides was performed with partial sternotomy. Our calculated incidence for chylothorax with total thyroidectomy and neck dissection was 1.85 %; for a thoracic approach the calculated incidence was 7.3 %.

**Conclusions:**

There are no reports of chylothorax after thyroidectomy without at least a left lateral neck dissection due to advanced thyroid cancer and/or sternotomy due to the thyroid size. The extension of thyroid surgery seems to be the main risk factor in developing chylothorax either through direct surgical trauma or through increased intraductal pressure after thoracic duct ligation. An early diagnosis of chylothorax may avoid severe metabolic or cardiopulmonary complications.

## Background

The most common complications after total thyroidectomy are hypoparathyroidism with hypocalcemia, hematoma and recurrent laryngeal nerve injury [1–3]. Chylous leak in the neck is a very rare complication after thyroidectomy. The risk of this complication increases when the thyroid surgery is associated with neck dissection (ND) (0.5 % risk after less than total thyroidectomy with central ND, 0.8 % after total thyroidectomy and central ND, 5.1 % after total thyroidectomy and lateral ND on one side, 6.2 % after total thyroidectomy and lateral ND on both sides) [4–6]. Chylothorax after thyroidectomy appears to be extremely rare and is reported only anecdotally. Although the cervical chyle leak can be explained by lateral ND, the risk factors and determinants of a chylothorax after thyroidectomy remain unknown.

Anatomically, the thoracic duct extends prevertebrally from the cisterna chyli (2nd lumbar vertebra), first from the right side, then from near the thoracal vertebra 4 on the left side, finally curving posteriorly to end in the left venous angle in the neck. However, the termination of the thoracic duct into the neck venous system is highly variable [7, 8]. It can terminate on the left side in the internal jugular vein (46 %), in the jugulo-venous angle (32 %), subclavian vein (18 %), external jugular vein, vertebral vein, transverse cervical vein, brachiocephalic vein, suprascapular vein, innominate vein, and in the right internal jugular vein (4 %) [9]. The right lymphatic duct is located at the medial border of the anterior scalene muscle, and its course is similar (although shorter) compared to the left side. A practical point worth remembering is that the duct on both sides lies on the fascia overlying the scalene muscles, and therefore far more susceptible to injury than the phrenic nerve, which lies under the fascia, when stripping away fatty and lymphatic tissue during surgery [10].

Experiencing a chylothorax case in our clinic after total thyroidectomy with central and lateral ND led us to systematically assess the frequency and determinants of this complication and investigate whether chyle leak was directly associated with resection of the thyroid gland.

We hypothesized that total thyroidectomy was associated with postoperative chylothorax, leading us to conduct a systematic review of the literature.

## Case report

A 54-year-old male patient with metastatic papillary thyroid carcinoma on his left side underwent total thyroidectomy with a bilateral central and lateral ND on the left side. Intraoperatively, the thoracic duct was identified and ligated. On the second postoperative day (POD), the patient complained of dyspnea, abdominal pain and slight dysphagia. Laboratory findings were normal except for hypocalcemia (ionized calcium: 1 mmol/l). Physical examination revealed a left vocal cord paralysis and cervical swelling with drainage of a sanguinary fluid from the left cervical drain. Imaging of the neck revealed a cervical hematoma on the left side, which was evacuated on POD 3. Low blood oxygen saturation during revision surgery was noted and a postoperative thoracic CT scan showed a subsegmental lung embolism on the right side as well bilateral, but predominantly right sided pleural effusions. In addition to standard unfractionated heparin therapy, thoracocentesis was performed on the right side, yielding chylous fluid. A triglyceride measurement in the fluid confirmed the diagnosis of a chylothorax. The patient underwent bilateral thoracic drainage and total parenteral nutrition was started (for a total of 18 days). After 14 days, an overlapping enteral medium chain triglyceride diet was installed. The patient’s condition improved steadily with decreasing thoracic effusions; the left thoracic drain was removed on POD 19 and the right one on POD 20.

Due to anticoagulation therapy, a second neck hematoma developed and was evacuated on POD 7. The patient was discharged on POD 24 and received ablative radioiodine therapy several weeks later.

## Methods

The search strategy was designed by GM and VM. We systemically reviewed the complete literature by searching PubMed databases up to April 2014 using the terms *thyroid surgery, thyroidectomy*, *chyle*, *chylothorax*, *and complication* (electronic search: thyroid surgery [tiab] OR thyroidectomy [mh]) AND (chyle [tiab] OR chylothorax [tiab] OR complication [tiab]). We did not restrict our search by patient age, diagnosis or publication date. Additionally, we performed a hand search of papers cited in the eligible articles. Additional reports were found through personal communication. Efforts were made to include non-English papers written in Italian, Spanish, French and German. If the published data were unclear, attempts were made to contact the authors. This review was conducted following PRISMA-Guidelines [11].

All abstracts were screened by two independent reviewers (VM, JP). Articles were selected using pre-determined criteria (Appendix). Papers were excluded if they did not include thyroid surgery or did not report chylothorax as an outcome. We did not restrict our inclusion criteria based on the type or quality of the clinical studies, because we suspected that the number of studies would be low. However, the overall methodological quality and risk of bias for all included articles was assessed and reported (Table 1). The full text was screened for eligibility by two independent reviewers (VM, JP) and verified by a third reviewer (GM), addressing any disagreements regarding article eligibility. Differences were resolved by discussion. We only included papers describing chylothorax as a complication after thyroidectomy. If there was uncertainty as to whether thyroid surgery has been performed, patients were excluded. One cohort study [12] included 3 patients from a previously published case report [13]. We included both papers to obtain additional information.

**Table 1:** Study type, patients’ characteristics and type of surgery

Author, Year	Case no.	Study type/Quality/Bias	Incidence	Age (years)/Gender	Diagnosis	Type of thyroid surgery	Neck Dissection		Intervention on thoracic duct
							Side	Type	
Har-El, 1985 [27]	1	CR/Level 5/report. + publ. bias	N/A	34/f	PTC	TT	Left	Centr. + lat.	ID + LIG
Jabbar,1995 [7]	2	CR/Level 5/report. + publ. bias	N/A	35/f	PTC	TT	Bilat.	Centr. + lat.	ID + LIG
Lifante, 2007 [17]	3	RCS/Level 3/select. Bias	2/52	N/A	N/A	TT + ST	N/A	N/A	N/A
	4			N/A	N/A	TT + ST	N/A	N/A	N/A
Bae, 2007 [25]	5	CR/Level 5/report. + publ. bias	N/A	46/f	PTC	TT	Left	Centr. + lat.	no ID
	6			47/f	PTC	TT	Left	Centr. + lat.	no ID
Tsukahara, 2007 [22]	7	CR/Level 5/rep. + publ. bias	N/A	72/f	PTC	TT	Left	Centr. + lat.	ID + LIG
Roh, 2008 [15]	8	PCS/Level 2/no bias	1/82	63/m	PTC	TT	Left	Centr. + lat.	no ID
Paliogiannis, 2009 [16]	9	RCS/Level 3/selection Bias	2/94	68/f	ATC	TT + partial ST	Bilat.	Centr. + lat.	no ID
	10			N/A	PTC	TT + partial ST	Bilat.	Centr. + lat.	no ID
Han, 2009 [20]	11	CR/Level 5/report. + publ. bias	N/A	42/f	MTC	TT	Bilat.	Centr. + lat.	ID + LIG
Khurana, 2009 [23]	12	CR/Level 5/report. + publ. bias	N/A	17/f	TC	TT	Bilat.	Centr. + lat.	N/A
Tallón-Aguilar, 2010 [26]	13	CR/Level 5/report. + publ. bias	N/A	38/f	PTC	TT	Left	Centr. + lat.	N/A
Li, 2013 [12]	14	RCS/Level 3/no bias	5/242	48/f	PTC	TT	Left	Lat.	ID + LIG
	15			65/f	PTC	TT	Left	Centr. + lat.	ID + LIG
Tian, 2012 [13] Li, 2013 [12]	16^a^	CR/Level 5/report. + publ. bias		40/f	PTC	TT	Bilat.	Centr. + lat.	ID + LIG
	17^a^			38/f	PTC	subT	Bilat.	Centr. + lat.	ID + LIG
	18^a^			31/f	PTC	subT	Bilat.	Centr. + lat.	ID + LIG
Runge, 2014 [21]	19	CR/Level 5/report. + publ. bias	N/A	40/f	PTC	TT	Bilat.	Centr. + lat.	ID + LIG
Our case	20	CR/Level 5/report. + publ. bias	N/A	54/m	PTC	TT	Left	Centr. + lat.	ID + LIG

*ATC* anaplastic thyroid cancer; *Bilat.* bilateral; *Centr.* central; *CR* case report; *f* female; *ID* identification; *LIG* ligation; *lat.* lateral; *m* male; *MTC* medullary thyroid cancer; *N/A* not available; *PCS* prospective cohort study; *PTC* papillary thyroid cancer; *report. Bias* reporting bias; *publ. bias* publication bias; *RCS* retrospective cohort study; *ST* sternotomy; *subT* subtotal thyroidectomy; *TC* thyroid cancer not specified; *TT* total thyroidectomy

^a^3 cases of Li 2013 [12] were previously published as case reports in Tian 2012 [13]

Data extraction was performed independently by two authors (VM, JP), and differences were resolved by consensus with input from two additional authors (RG, GM). Information abstracted from each article included study type, patient demographics, diagnosis, incidence of chylothorax (papers without selection bias), differences in thyroid surgery, type of ND (central/lateral), intraoperative handling of the thoracic duct, timing of chylothorax diagnosis, diagnostics, duration and treatment of chylothorax including thoracic drainage.

All available data from case series or case reports were considered for descriptive analysis of patients. The incidence of chylothorax was calculated from unbiased studies. Some articles did not provide complete information on individual subjects. In one case of malignant disease without specific histology we used the term thyroid cancer. In 2 patients, the diagnosis was not further specified.

Descriptive statistics were calculated using SPSS (IBM SPSS. Version 21. Armonk, USA; 2012). Cohen’s kappa coefficient was used to assess the inter-rater agreement.

## Results

**Fig. 1:**
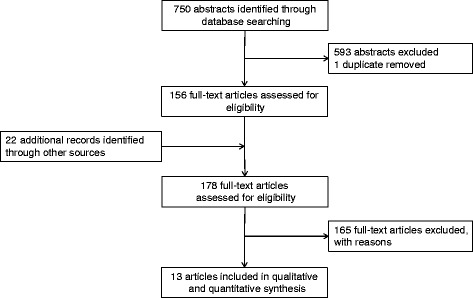
Flow diagram of the search and review process

Figure 1 illustrates the search and review process. Our search identified 750 unique citations, of which 593 were excluded at the abstract review level (inter-rater reliability κ = 0.553). The most common reason for exclusion was “no chylothorax”. After a full-text search of 178 manuscripts identified by electronic or hand search, 13 publications (9 case reports, 3 retrospective and 1 prospective cohort study) were included in the final review (inter-rater reliability κ = 0.809). Tables 1 and 2 show the details of the different article types [14], patients characteristics, type of surgery and individual treatment strategies. In total, 20 patients, including our own, were retained. Table 3 summarizes all of the results.

**Table 2:** Symptoms, diagnostics and treatment of chylothorax

Author, Year	Case no.	Postoperative Symptoms	Side of Chylothorax	Cervical Chyle Leak (Side)	POD of Chylothorax	Imaging (Thorax)	Treatment			
							Thorax drain	Duration of drainage (days)	Diet^c^	Surgical Revision
Har-El, 1985 [27]	1	dyspnea, chest pain	Bilat.	no	3	X	Right^a^	<1	yes	no
Jabbar,1995 [7]	2	dyspnea, chest pain	Bilat.	no	4	X	Bilat.	<1	no	no
Lifante, 2007 [17]	3	N/A	N/A	N/A	N/A	N/A	RS	N/A	N/A	yes
	4	N/A	N/A	N/A	N/A	N/A	RS	N/A	N/A	no
Bae, 2007 [25]	5	dyspnea, chest discomfort	Bilat.	no	7	X + CT	Bilat.	N/A	yes	no
	6	dyspnea	Bilat.	no	3	X + CT	Bilat.	3	yes	no
Tsukahara, 2007 [22]	7	dyspnea	Bilat.	no	2	X	Bilat.	2	yes	no
Roh, 2007 [15]	8	N/A	Bilat.	yes (Left)	25	N/A	Bilat.	20	yes	yes^d^
Paliogiannis, 2009 [16]	9	N/A	Left	N/A	N/A	N/A	Left	20	yes	no
	10	N/A	Left	N/A	N/A	N/A	Left	N/A	N/A	yes
Han, 2009 [20]	11	dyspnea, chest pain	Bilat.	no	8	X	Left^a^	5	yes	no
Khurana, 2009 [23]	12	dyspnea, chest discomfort	Bilat.	no	1	X	Bilat.	9	yes	yes^e^
Tallón-Aguilar, 2010 [26]	13	dyspnea	Bilat.	N/A	3	X	Left^a^	N/A	yes	yes^f^
Li,2013 [12]	14	chest discomfort	Bilat.	yes (N/A)	2	N/A	Bilat.	N/A	yes	no
	15	dyspnea, chest discomfort	Bilat.	yes (N/A)	8	N/A	Bilat.	N/A	yes	no
Tian, 2012 [13] Li, 2013 [12]	16^g^	chest discomfort	Bilat.	yes (Left)	2	X	Bilat.	15	yes	yes
	17^g^	dyspnea, chest discomfort	Left	no	4	X	Left	4	yes	no
	18^g^	dyspnea, chest discomfort	Bilat.	yes (N/A)	3	US	Bilat.	7	yes	no
Runge, 2014 [21]	19	dyspnea, chest discomfort	Bilat.	no	2	CT	Bilat.	7	yes^b^	yes^e,f^
Our case	20	dyspnea, abdominal pain	Bilat.	yes (Left)	3	CT	Bilat.	17	yes	no

*Bilat.* bilateral; *X* standard X-ray; *CT* computertomography; *N/A* not available; *POD* postoperative day; *RS* retrosternal; *US* ultrasound

^a^unilateral thoracic drainage, but bilateral chylothorax described

^b^patient received additionally octreotide administration

^c^consisting of enteral diet rich in medium-chain triglycerides and/or total parenteral nutrition

^d^gelfoam packing, fistula ligation and pectoralis major muscle flap transfer

^e^wound exploration without identification of leak site

^f^thoracic duct ligation +/− sealing with biological glue

^g^3 cases of Li 2013 [12] were previously published as case reports in Tian 2012 [13]

**Table 3:** Summary of demographic, clinical and therapeutic characteristics of all 20 reported patients

Characteristics	N of cases	Mean/SD/Percentage/Range
*Age [years]*	17^a^	mean 45.7; SD: 14.6; range: 17-72
*Gender*	17^a^	
Female	15	88.2 %
Male	2	11.8 %
*Diagnosis*	20	
Cancer	18	90.0 %
PTC	15	
MTC	1	
ATC	1	
TC	1	
N/A	2	10.0 %
*Surgery*	20	
TT + ND	14	
Sub T + ND	2	
TT + partial ST + ND	2	
TT + ST	2	
*Thoracic duct reported*	16^a^	
ID + LIG	11	69.0 %
no ID	5	31.0 %
*Neck Dissection*	18	
Bilat.	9	
Left	9	
*Type of Neck Dissection*	18	
Centr. + Lat.	17	
Lat.	1	
*Time of diagnosis (POD)*	16^a^	mean 5, SD: 5.75, range: 1-25
*Side of Chylothorax*	18^a^	
Bilat.	15	83.0 %
Left	3	17.0 %
*Duration of drainage (days)*	13^a^	mean: 8.5, SD: 7, range: 1-20
*Treatment after thoracic drainage*	20	
Conservative with diet alone	12	
Combined conservative and surgery	5	
Surgery only	2	
N/A	1	

*ATC* anaplastic thyroid cancer; *Bilat.* bilateral; *Centr.* central; *ID* identification; *LIG* ligation; *Lat.* lateral; *MTC* medullary thyroid cancer; *N/A* not available; *POD* postoperative day; *PTC* papillary thyroid cancer; *SD* standard deviation; *ST* sternotomy; *TC* thyroid cancer not specified; *sub T* subtotal thyroidectomy; *TT* total thyroidectomy

^a^20 screened subjects; not all reports provided complete clinical information

The calculated mean age was 45.7 years. The male: female ratio was 1:7. Not all articles reported the exact reason for thyroidectomy; the most common diagnosis was papillary thyroid cancer. In two patients who had a sternotomy, the diagnosis was not specified. There were no reports of chylothorax in patients undergoing thyroidectomy alone. The calculated unbiased incidence of chylothorax in thyroid surgery with a cervical approach was 1.85 % (6/324 [12, 15]) and with a thoracic approach, the incidence was 7.3 % (4/55 [16, 17]). Eighteen patients received a total or subtotal thyroidectomy at least with a lateral ND. Two of the patients underwent an additional partial sternotomy (manubriotomy) to improve accessibility. In the remaining two patients, a total sternotomy was performed. In 11 of 16 patients mentioning the thoracic duct, the duct was intraoperatively ligated. In the other 5 patients, the duct was not identified.

Overall, 18 patients underwent a lateral ND, and 2 of them experienced additional partial sternotomy. In the 2 remaining patients, a sternotomy was performed without a ND. In 9 patients, the lateral ND was bilateral. All reported patients with ND except one had an additional central ND. Nine of the 18 patients (50 %) underwent a bilateral ND; the remaining 9 patients received only a left-sided ND. There were no reports of exclusively right-sided ND.

The mean time until chylothorax diagnosis after surgery was 5 days (range: 1–25 days). Fifteen of the 18 patients (83 %) in articles that mentioned the location of the chylothorax (right or left) were bilaterally affected. There were no cases of right sided chylothorax alone. Initial manifestations of chylothorax were dyspnea, bilateral chest pain and chest discomfort. In our case, dyspnea, abdominal pain and low blood oxygen saturation were described. Imaging (standard thoracic X-ray, CT scan and ultrasound) revealed pleural effusions and subsequent fluid analysis confirmed the presence of chyle. Six out of 15 reported patients (40 %) suffered from a previous cervical chyle fistula; in five of them the thoracic duct had been previously ligated or preserved during thyroidectomy.

All 20 patients underwent thoracic drainage as an initial diagnostic and therapeutic procedure. The mean duration of drainage was 8.5 days (range: 1–20). Twelve of 16 patients (75 %) in articles that reported nutrition strategy were successfully treated conservatively with a median-chain triglyceride diet or/and total parenteral nutrition. Octreotide infusion was additionally administered to one patient. In total, 7 patients underwent an additional operation; five needed surgical revision despite dietary restriction. Surgical revision alone is described in two patients. Both patients most likely underwent a second sternotomy. In one of the 7 patients, chyle leakage was controlled following fistula ligation with gelfoam packing and muscle flap transfer from the pectoralis major muscle on POD 27. There were also 2 cases requiring an additional operation where no identification of the thoracic duct leak site could be made by wound exploration.

## Discussion

There were no reports about chylothorax and isolated thyroidectomy found. The risk factors for chylothorax in thyroid surgery include a concomitant, at least left-sided lateral ND and/or sternotomy needed for advanced thyroid cancer, extensive surgery due to the thyroid size or intrathoracic localization.

Because of the rarity of chylous leak in the neck after cervical surgery, the incidence reported in the literature varies, ranging from <1 % to 6.2 % [5, 6, 12, 18]. However, the rate of chyle fistula is dependent of the extent of surgical procedure in the neck [19]. The reported incidence of chylothorax after thyroidectomy with lateral ND ranges from 1.2 % [15] to 2.07 % [12] and increases to 3.8 % [17] with thoracic approach and partial or total sternotomy. Considering our review data, the calculated incidence of chylothorax in thyroidectomy with cervical approach (thyroidectomy and ND) is 1.85 % [12, 15], which is consistent with the literature. However, the calculated incidence of chylothorax in thyroidectomy with thoracic approach (thyroidectomy and sternotomy) is much higher than reported (7.3 %) [16, 17]. This incidence may be overestimated, since our calculations are based on a few cohort studies with potential bias due to methodology.

The typical patient suffering from this complication is female, middle aged (41 years), has a diagnosis of thyroid cancer and undergoes thyroidectomy with additional lateral ND. Nearly all articles in our systematic review described the intraoperative situs and reported whether the thoracic duct was identified or ligated. However, neither preservation nor ligation prevented the formation of a chylothorax [7, 12, 20–22]. It appears that the identification of the thoracic duct in cervical surgery does not play a major role in avoiding a cervical or thoracic chyle leak. In one case, the thoracic duct could not be identified in a second-look operation, but the chylothorax resolved [23].

Two pathomechanisms for acquired (iatrogenic) chylothorax are mentioned in the literature [13, 20, 21, 23–25]: (1) direct leakage due to traumatic injury during surgery, a notable amount of chyle leaks directly from the base of the neck into the mediastinum [23, 24, 26], (2) increase of backward hydrostatic pressure in the thoracic duct after duct ligation [27], causing the intraluminal pressure on the duct to increase to the point at which a non-traumatic extravasation of chyle is created or a secondary rupture of the thoracic duct occurs [7, 24]. In both cases the chyle penetrates into the pleura as a result of tissue maceration and inflammatory reaction. These pathomechanisms are supported by the negative pressure in the thoracic cage during inspiration, leading to the extravasation through the dilated intrapulmonary lymphatic vessel and entrance of chyle into the pleural space, resulting in chylothorax [25]. There is no direct injury to the pleura, explaining the absence of pneumothoraces in any reported case in the literature [25]. The extravasation point is in the mediastinum and not in the neck, which can explain the absence of external chylorrhoea. Both mechanisms may act synergistically [20]. In the retained articles, both pathomechanisms seem to occur. Some patients had a previous cervical chyle leak; others showed a chylothorax after ligation of the thoracic duct. Five of the patients with a ligated thoracic duct had a cervical chyle leak, which is not in line with the common known pathomechanisms. We presume that an injury or an incomplete ligation of the thoracic duct might occur more often than expected [23, 24, 26]. Chylothorax was most often diagnosed around the 5th postoperative day, which is too late to result from direct injury of the duct. Because the pathomechanisms are still hypothetical, we suggest using a new and more appropriate nomenclature such as ‘early acquired chylothorax’ for leaks due to direct neck trauma (injury and/or ligation of the duct) and ‘late acquired chylothorax’ for delayed leaks without preceding trauma of the thoracic duct.

Diagnosis of chylothorax is based on clinical findings including postoperative respiratory symptoms (dyspnea, chest discomfort or chest pain) and radiological confirmation of large pleural effusions using standard chest X-ray, CT scan or ultrasound. The most common imaging tool was standard thoracic X-ray. Dyspnea could also be a result of other conditions, such as bilateral recurrent nerve paralysis, hypocalcemia or lung embolism [2, 6, 28], which is the reason why this rare complication might be initially missed. After diagnostic thoracocentesis, the analysis of the milky fluid confirms the existence of chyle [7, 20–22, 25, 26].

Complications mainly consist of loss of fluid, electrolytes, proteins, fat, fat-soluble vitamins and lymphocytes, predominantly the T-cell variety. Therefore, chyle leakage may lead to metabolic imbalances, nutritional deficiency and infections due to immunodeficiency [22, 29]. Chyle in the pleural space can cause cardiopulmonary compromise because of compression of the lungs, leading to a mediastinal shift with distortion of the great vessels [20].

The management of chylothorax is not clearly established. Dietary restriction alone was one of the main pillars in the treatment (2/3 of the patients). One third of the patients underwent at least a thoracic drainage. Twenty-five percent underwent a combination of conservative and surgical treatment. A conservative treatment is the most accepted therapeutic strategy. The first step is a diagnostic and therapeutic thoracocentesis with relief of respiratory symptoms. [8, 29, 30] Thoracic drainage, although invasive, is considered an important step for a successful conservative treatment. Additional therapy consists of nutritional modifications with the aim to reduce chyle production and to prevent metabolic complications. Dietary options include (1) complete fasting for enteral rest and administration of total parenteral nutrition or (2) a high protein-low fat diet supplemented with middle-chain triglycerides [31]. An additional, less used therapeutic option to reduce chyle flow is octreotide, a synthetic somatostatin analogue [31–33]. Indications for a surgical intervention (duct repair) include persistent fistulae in spite of conservative treatment, significant leakage or associated complications. Some authors propose a limit in quantity (>1 liter per day for 5 days) or time of drainage (>2 weeks) for conservative treatment, but there is no international consensus [8, 24–26, 34, 35]. An earlier surgical intervention (between the 3rd and the 7th day after chyle leak diagnosis) may shorten the length of hospital stay in young adult patients who have a lower risk profile for additional postoperative complications [35]. In our review, two patients underwent an additional operation due to the increasing amount of thoracic discharge (up to 2 liters per day). In one patient, the fluid effusion decreased very slightly [15]. The youngest patient, who was 17-years old, received a revision on the 3rd postoperative day despite being hemodynamically stable [23]. Surgical revisions included ligation, biological glue and even a muscular flap for duct repair [15, 23, 26]. In at least 2 out of 7 re-operated patients, the leakage point was not found [21, 23] and conservative therapy was continued with resolution of the chylothorax [20, 22]. In view of these reports, it remains unknown if surgical duct repair shortens the duration of hospital stay or reduces potential complications, because 90 % of the operated patients received an initially conservative treatment as well. The decision for surgical intervention depends on the condition of the patient and the amount of leakage.

The limitation of our review is that it covers a small number of included publications mainly consisting of case reports and retrospective cohort studies. The number of reported cases might be underestimated (publication bias), because chylothorax may not be considered as a direct complication of thyroidectomy and may be attributed to secondary interventions such as ND or sternotomy. Some papers focused on other topics than chylothorax, a reason why reported data were often incomplete or missing.

## Conclusions

A chylothorax in thyroid surgery is a very rare complication and there is no report of chylothorax after a thyroidectomy without associated surgical procedures such as ND or sternotomy. Both lateral, at least left-sided ND and sternotomy are the main associated risk factors in developing chylothorax, increasing its incidence to 1.85 % and 7.3 %, respectively. A chylothorax only on the right side has not yet been described. Chylothorax is not implicitly associated with a synchronous cervical chyle leak.

Even though very rare, chylothorax should still be considered as a potential postoperative complication in patients with respiratory symptoms after thyroid surgery associated with a lateral ND and/or sternotomy.

## Appendix

**Table 4:** Exclusion coding schema for abstract reviews

1	Other Language than English, Spanish, Italian, French or German	Manuscript is not in English, Spanish, Italian, French or German
2	Animal-surgery	Only human patient data
3	No thyroid surgery	No reasonable prospect that the study includes data about thyroid surgery
4	Chylothorax before surgery	No reasonable prospect that chylothorax is a result of the thyroid surgery
5	No reasonable prospect that the final manuscript contains information about complications with possible chylothorax	No reasonable prospect that the study deals with chylothorax

**Table 5:** Exclusion coding schema for full-text reviews

1	Other Language than English, Spanish, Italian, French or German	Manuscript is not in English, Spanish, Italian, French or German
2	Animal-surgery	Only human patient data
3	No thyroid surgery	No reasonable prospect that the study includes data about thyroid surgery
4	Chylothorax before surgery	No reasonable prospect that chylothorax is a result of the thyroid surgery
5	No chylothorax	No reasonable prospect that the study deals with chylothorax
